# Short-Term Efficacy and Safety Outcomes of Brolucizumab in the Real-Life Clinical Practice

**DOI:** 10.3389/fphar.2021.720345

**Published:** 2021-11-04

**Authors:** Andrea Montesel, Claudio Bucolo, Ferenc B. Sallo, Chiara M. Eandi

**Affiliations:** ^1^ Department of Ophthalmology, University of Lausanne, Fondation Asile des Aveugles, Jules Gonin Eye Hospital, Lausanne, Switzerland; ^2^ Department of Biomedical and Biotechnological Sciences, School of Medicine, University of Catania, Catania, Italy

**Keywords:** brolucizumab, anti-vascular endothelial growth factor, intravitreal route, neovascular age-related macular degeneration, retina, choroid

## Abstract

To report the early efficacy and safety outcomes of treatment with intravitreal injections of brolucizumab (IVT-B) in patients presenting neovascular age-related macular degeneration (nAMD) in a tertiary clinical setting. A retrospective case series of patients that received IVT-B with a minimum of two injections performed and at least 4 weeks of follow-up after last injection. Nineteen eyes of 19 patients were included. The number of IVT-B performed for the whole cohort was 58 injections; the mean number of IVT-B per patient was 3.0 ± 1.0 (range 2–6); the mean follow-up time was 14.4 ± 9.0 weeks. Mean baseline best-corrected visual acuity was 0.4 ± 0.4 logMAR and at the last follow-up was 0.4 ± 0.6 logMAR (*p* = 0.778). All eyes showed a reduction in retinal thickness, with the central macular thickness being 470 ± 151 μm at baseline and 360 ± 144 μm at the last follow-up (*p* = 0.001). Intra-retinal fluid was present at baseline in 12 eyes (63%) and in three eyes (16%) at the last follow-up (*p* = 0.065). Sub-retinal fluid was present at baseline in 17 eyes (89%) and at the last follow-up in three eyes (16%, *p* = 0.011). Pigment epithelium detachment was apparent in the 16 eyes (84%) at baseline and was still present in 14 eyes (73%, *p* = 0.811). One adverse event of intraocular inflammation was reported. In conclusion, our short-term experience showed that brolucizumab was highly effective in restoring the anatomy and in stabilizing the visual acuity of eyes with nAMD. Its safety profile should be evaluated carefully and needs further investigations.

## Highlights

Brolucizumab, the newest agent approved in clinical use for the treatment of neovascular age-related macular degeneration, was designed by grafting the complementarity-determining regions of a novel anti-VEGF-A antibody onto a human single-chain antibody fragment. Lately, the American Society of Retinal Specialists highlighted some Adverse Drug Reactions in patients receiving brolucizumab. The present study reported short-term effective anatomical and functional outcomes of brolucizumab in a real-world practice, which confirm results from randomized clinical trials. These data are important for the ophthalmic community and the pharmacovigilance system, particularly for the safety profile. The safety profile of brolucizumab needs further investigations and signs of intraocular inflammation should be evaluated with careful patient monitoring.

## Introduction

Age-related macular degeneration (AMD) is the most common cause of irreversible vision loss in people aged 55 years and older in western industrialized countries (Reibaldi et al., 2016). Neovascular AMD (nAMD) accounts for most cases of AMD-related severe vision loss (Solomon et al., 2019). Intravitreal (IVT) injections of anti-vascular endothelial growth factor (VEGF) agents are currently the gold standard treatment of nAMD (Li et al., 2020). In general, the goals of anti-VEGF therapy in neovascular AMD are to achieve excellent functional visual acuity and maintain a dry macula on clinical and OCT examination. The duration of VEGF suppression appears to vary between drugs as well as with individualized patient responses. On this regard, new effective drugs or innovative intravitreal nano-systems represent two strategies to reduce the injection burden (Conti et al., 1997). Brolucizumab is a newly available anti-VEGF agent that has recently been approved for intraocular use by the United States Food and Drug Administration (FDA) and the European Medicines Agency (EMA), based primarily on the results of two large phase three, multicenter, active-controlled, randomized, double-masked trials, HAWK and HARRIER (Dugel et al., 2020). Brolucizumab (Beovu^®^, Novartis Pharma AG, Basel, Switzerland) is a low molecular weight (26 kDa versus 48 kDa of ranibizumab and 115 kDa of aflibercept), single-chain antibody fragment that targets all forms of VEGF-A with high affinity (Ricci et al., 2020). The results of the registration trials of brolucizumab were promising, showing gains in visual acuity that were non-inferior to aflibercept and better anatomical outcomes than aflibercept, with a similar safety profile (Sharma et al., 2020). Brolucizumab showed a potential extension of the dosing regimen to 12 weeks intervals, reducing the burden of the treatment, on both patients and physicians. Nevertheless, an increasing number of unforeseen post-marketing adverse events (AEs) following brolucizumab treatment have been reported as intraocular inflammation (IOI), retinal vasculitis and/or retinal artery occlusion associated with severe vision loss (Monés et al., 2020); so there is a rising concern about its clinical use in the ophthalmology community (Rosenfeld et al., 2020). Clinical data regarding the use of intravitreal injections of brolucizumab (IVT-B) outside the above-mentioned clinical trials are still limited. To our knowledge, there are only a few brief reports that investigated the real-world efficacy and safety of brolucizumab (Avayon et al., 2020; Bulirsch et al., 2021; Maruko et al., 2021; Sharma et al., 2021), thus our investigation aims to report our real-life experience with this new anti-VEGF agent in a cohort of nAMD patients.

## Materials and Methods

### Study Population

This is a retrospective, observational, monocentric study at the Jules Gonin Eye Hospital. Clinical records of nAMD patients treated with IVT-B in routine clinical practice at our Institution from March to December 2020 were reviewed. Inclusion criteria were a diagnosis of nAMD with any type of choroidal neovascularization involving the foveal region, a minimum of two injections, including the loading dose, and 4-weeks follow-up following the last IVT-B. Exclusion criteria were the presence of macular diseases other than nAMD, and a history of intraocular inflammation. Treatment-naïve nAMD eyes as well as eyes already under treatment with anti-VEGF intravitreal injection for active choroidal neovascularization (CNV) secondary to nAMD were enrolled. For treatment-naïve eyes, the decision of treatment with IVT-B was proposed to the patient following an extensive discussion of the risks and benefits involved. For patients already receiving IVT injections, the decision of switching to brolucizumab was proposed by the retina specialist based on active exudation persisting after at least three IVTs of other anti-VEGF agents (ranibizumab or aflibercept). The first IVT-B was performed 1 month after the last ranibizumab and 2 months after the last aflibercept. The presence of refractory active exudation was assessed by a senior retina specialist (C.M.E) on the basis of OCT qualitative features suggestive of exudative disease activity following previously published guidelines (Schmidt-Erfurth et al., 2014; Spaide et al., 2020). All patients provided written informed consent before the IVT-B, as usual procedure in clinical care.

Prior to the first IVT-B, all patients underwent best-corrected visual acuity (BCVA) measurement using an Early Treatment Diabetic Retinopathy Study (ETDRS) chart, intraocular pressure (IOP) measurement, a complete slit lamp biomicroscopy examination, fundus ophthalmoscopy following pupil dilation with 1% tropicamide eye drops, and optical coherence tomography (OCT) (Heidelberg Spectralis, Heidelberg Industries, Heidelberg, Germany) imaging.

In the case of treatment-naïve nAMD eyes, fluorescein (FA) and indocyanine green (ICGA) angiography were also performed (Heidelberg Spectralis, Heidelberg Industries, Heidelberg, Germany). Follow-up visits were monthly for the first 3 months, and bimonthly (q8) or every 3 months (q12) subsequently, accordingly to the treatment interval. At each follow-up visit, patients underwent BCVA and IOP measurements, slit-lamp anterior segment and fundus biomicroscopic examinations and OCT imaging.

### Treatment Protocol and IVT Injections

IVT-B treatment was scheduled according to the label registered with SwissMedic, the Swiss Agency for Therapeutic Products Brolucizumab (Beovu®, 2020) (https://www.swissmedic.ch/swissmedic/en/home/about-us/publications/public-summary-swiss-par/public-summary-swisspar-beovu.html. Accessed 30 Nov 2020). An initial loading dose of three monthly IVT-B injections was scheduled. Safety evaluations were conducted at every visit, with special regard to signs of IOI. The symptoms of IOI were explained to each patient and they were advised to contact the clinic immediately, should any of these symptoms (such as floaters, blurred vision, metamorphopsia, scotoma, visual field defects) appear during follow-up. Treatment intervals following the loading dose were determined by a senior retinal specialist (CME) based on clinical evaluation of the anatomical and functional response to the first three doses and a q8 or a q12 interval was adopted as necessary, in accordance with the registration trials (Dugel et al., 2020).

IOI and uveitis reaction were graded according to the Standardization of Uveitis Nomenclature (SUN) Working Group guidelines (Nussenblatt et al., 1985; Jabs et al., 2005).

### Data Collection and Analysis

Collected data included the demographic characteristics and ophthalmic history of the patients, the number of previous intravitreal injections, functional (BCVA) and anatomical parameters, and postoperative complications. For non-treatment-naïve eyes, data just prior to the first IVT-B was considered the baseline, and the subsequent data after brolucizumab injections were included in the analysis. Therefore, in this study we analyzed BCVA at baseline before the IVT-B and at the last follow-up. Central macular thickness (CMT) was measured in OCT volume scans in the central subfield of an Early Treatment of Diabetic Retinopathy Study grid, centered on the fovea. Centering to the fovea and retinal layer segmentation during OCT examination were manually controlled and adjusted if needed for fixation or segmentation misalignments. The presence of intra-retinal fluid (IRF), sub-retinal fluid (SRF), and pigment epithelial detachment (PED) were recorded in OCT B-scans at baseline and at the last follow-up visit by two independent retinal specialists (CME and AM). A qualitative grading as resolution, reduction or increase of IRF, SRF, and PED from baseline to last follow-up was also performed based on the OCT B-scans by two independent retinal specialists (CME and AM). In case of disagreement, a third retina specialist (FS) graded the scans.

Statistical analysis was performed using SPSS software (Version 25.0, SPSS Inc., Chicago, United States). Data were analyzed with frequency and descriptive statistics for qualitative variables. The normality of data samples was assessed with the Kolmogorov–Smirnov test. Statistical comparison between baseline and the last follow-up data for continuous variables was performed using Student’s paired *t*-test with a 95% CI. Correlation for multiple testing was performed using the Bonferroni test. A two-tailed Fisher’s exact test was used to analyze the association between categorical variables with results statistically significant when associated with a *p*-value ≤ 0.05.

## Results

### Characteristic of the Study Population

**FIGURE 1:**
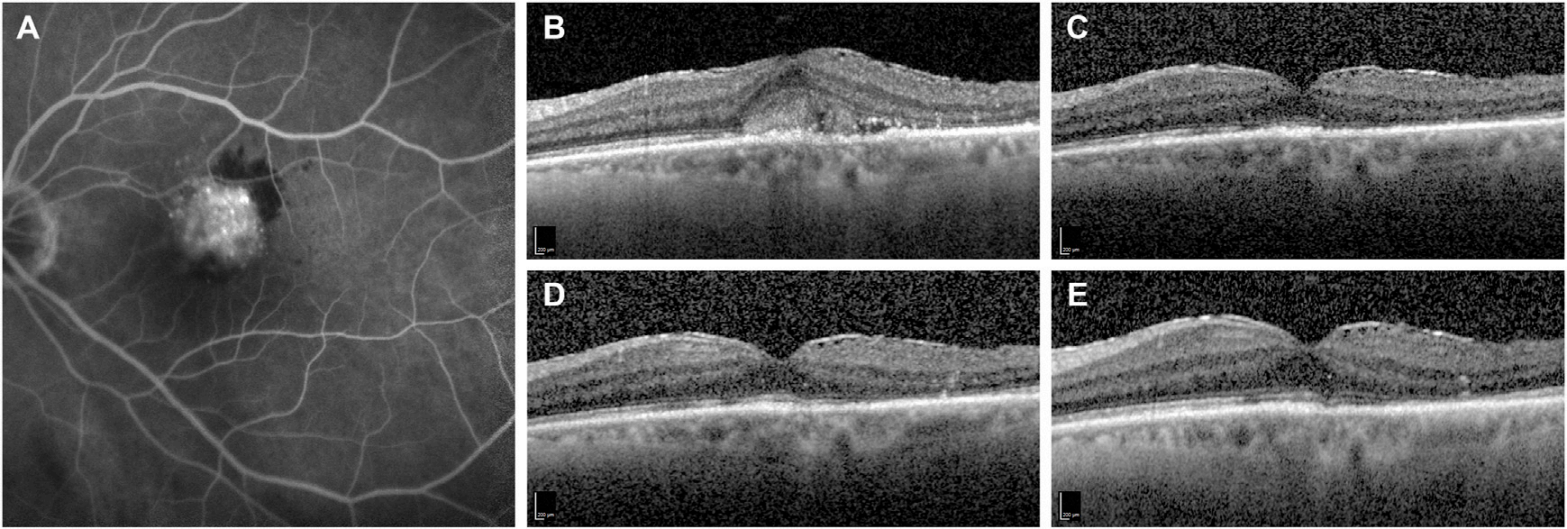
Representative case of a naïve patient presenting with choroidal neovascularization secondary to neovascular age-related macular degeneration treated with monthly intravitreal brolucizumab (IVT-B). **(A)** Baseline fluorescein angiography demonstrates the presence of a subfoveal type 2 choroidal neovascularization with adjacent subretinal hemorrhage. **(B)** Optical coherence tomography (OCT) B-scan at baseline reveals the presence of mild subretinal fluid (SRF) and subretinal hyperreflective material (SHRM) in the foveal area. An epiretinal membrane is also visible. **(C)** OCT B-scan 1 month after the first IVT-B shows the resolution of the SRF and the SHRM with reduction of the central subfield thickness from 375 to 295 µm. **(D)** OCT B-scans at 2-month and **(E)** at 3-month visit demonstrate the absence of recurrences and a dry macula after the loading dose. Best-corrected visual acuity improved from 20/100 to 20/50.

Nineteen eyes of 19 patients met the inclusion criteria and were included in the analysis. The baseline characteristics of the study population are summarized in Table 1. Fourteen patients were female (74%) and five males (26%), with a mean age of 78 ± 8.4 years. Four eyes (21%) were treatment naïve, while 15 eyes (79%) had been previously treated using other available anti-VEGF agents. In particular, 14 eyes received a total of 350 IVT injections of ranibizumab (mean 25.0 ± 29.5, range 3–98), and nine eyes received a total of 358 IVT injections of aflibercept (mean 39.7 ± 32.5, range 3–90). Over the study time, the total number of IVT-B injections performed was 58, with a mean of 3.0 ± 1.0 (range 2–6) injections per eye, and an average follow-up time of 14.4 ± 9.0 (range 4.0–35.8) weeks (Table 1). At anterior segment and fundus biomicroscopic examination, the study population presented several factors limiting the potential for visual recovery. In particular, four eyes (21%) presented geographic atrophy and/or retinal fibrosis affecting the central foveal area, three eyes (16%) presented a clinically relevant cataract, one eye (5%) was amblyopic and one eye (5%) presented an epiretinal membrane (Figure 1). Seven eyes (37%) were pseudophakic and one eye (5%) had received verteporfin photodynamic therapy (PDT) for nAMD 1 year prior to baseline. No other ocular comorbidity was reported and no topical treatment was used apart from lubricants. None of the patients had a previous history of intraocular inflammation.

**TABLE 1:** Characteristics of the Study Population.

Gender (n)	74% female (14), 26% male (5)
Mean age ± SD (Range)	78.0 ± 8.4 (63–92) Years
Naïve/Previously treated Eyes (n)	79% Eyes previously treated with IVT injections (15), 21% Treatment naïve eyes (4)
Previous IVT injections, Mean ± SD (Range) (Naïve patient excluded, n = 15)	47.2 ± 43.8 (4–139)
	14 eyes ranibizumab 25.0 ± 29.5 (3–98)
	9 eyes aflibercept 39.7 ± 32.5 (2–90)
Mean N° of IVT-B ± SD (Range)	3.0 ± 1.0 (2–6)
Mean follow-up time ± SD (Range)	14.4 ± 9.0 (4.0–35.8) Weeks

[SD, standard deviation; IVT, intravitreal; IVT-B intravitreal injection of brolucizumab].

### Functional and Anatomic Outcomes

The complete functional and anatomical parameters are summarized in Tables 2, 3.

**TABLE 2:** Functional and Anatomical Outcomes.

Outcome	Baseline	Last follow-up	*p*
Mean BCVA (logMAR) ± SD (Range)	0.4 ± 0.4 (−0.1–1.3)	0.4 ± 0.6 (−0.1–2)	0.778^a^
Mean CMT (µm) ± SD (Range)	470 ± 151 (235–802)	360 ± 144 (203–728)	0.001*^a^
Presence of IRF (n)	63% (12)	16% (3)	0.065^b^
Presence of SRF (n)	89% (17)	16% (3)	0.011*^b^
Presence of PED (n)	84% (16)	73% (14)	0.811^b^

[*p*, *p*-value; BCVA, best corrected visual acuity; LogMAR, logarithm of the minimum angle of resolution; CMT, central macular thickness; IRF, intra-retinal fluid; SRF, sub-retinal fluid; PED, pigment epithelial detachment].

* statistically significant.

a Student paired *t*-test.

b Two-tailed Fisher’s exact test.

**TABLE 3:** Analysis of the OCT parameters of exudation.

Baseline	Resolved	Reduced	Stable	Increased
IRF (n = 12)	75% (n = 9)	25% (n = 3)	0% (n = 0)	0% (n = 0)
SRF (n = 17)	82% (n = 14)	18% (n = 3)	0% (n = 0)	0% (n = 0)
PED (n = 16)	13% (n = 2)	31% (n = 5)	56% (n = 9)	0% (n = 0)

[OCT, optical coherence tomography; IRF, intra-retinal fluid; SRF, sub-retinal fluid; PED, pigment epithelial detachment].

Mean BCVA at baseline was 0.4 ± 0.4 [range from −0.1 to 1.3] logMAR [Snellen equivalent 20/50 (20/16—20/400)], and at the last follow-up was 0.4 ± 0.6 [range from −0.1 to 2] logMAR (20/50 [20/16—20/2000], *p* = 0.778, 95% CI = −0.15 to 0.11, post-hoc statistical power analysis 7.2%). Seven eyes (37%) gained at least one line of vision, nine eyes (47%) remained stable, and three eyes (16%) lost one line of vision.

**FIGURE 2:**
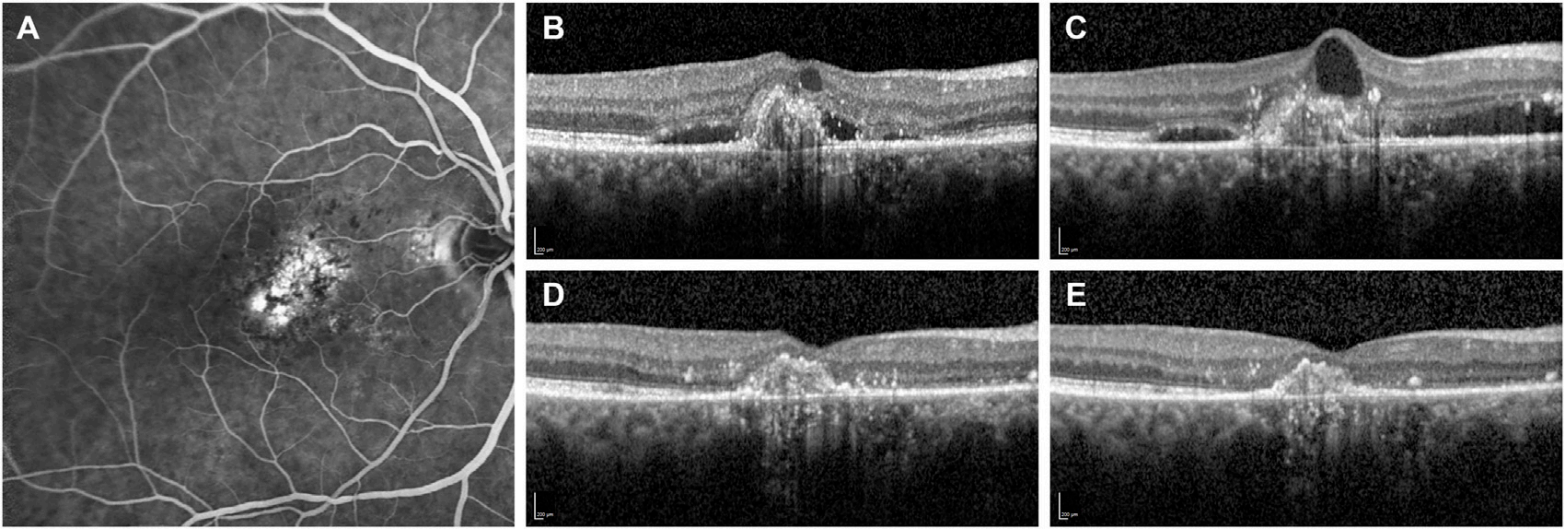
Fluorescein angiography and optical coherence tomography (OCT) B-scans of a patient with subfoveal choroidal neovascularization secondary to neovascular age-related macular degeneration. **(A)** Baseline fluorescein angiography demonstrates the presence of a subfoveal type 1 choroidal neovascularization. **(B)** OCT B-scan at the diagnosis shows a hypereflective subfoveal lesion with intra- and sub-retinal accumulation of fluid (IRF and SRF). **(C)** OCT B-scan at 13-month follow-up after a total of eight IVT injections of ranibizumab demonstrates the persistence of IRF and SRF. Therefore, the patient was switched to monthly intravitreal brolucizumab (IVT-B). **(D)** OCT B-scan 1-month visit after the first IVT-B reveals the complete resolution of IRF and SRF with a reduction of the central subfield thickness from 354 to 233 µm. **(E)** OCT B-scan at 3-month visit after the third IVT-B shows a dry retina and no recurrences are observed. Furthermore, the visual acuity improved from 20/125 to 20/80.

Concerning the anatomical outcomes, we observed an overall reduction of macular thickness from baseline in all 19 eyes. In particular, the CMT was 470 ± 151 µm (range 235–802) at baseline and 360 ± 144 µm (range 203–728) at last the follow-up (*p* = 0.001, 95% CI = 51.95–168.98, post-hoc statistical power analysis 94.8%). IRF was present in 12 eyes (63%) at baseline and in three eyes (16%) at the last follow-up. This change was not statistically significant (*p* = 0.065). SRF was present in 17 eyes (89%) at baseline and in three eyes (16%) at the last follow-up visit. This difference was statistically significant (*p* = 0.011). The OCT B-scan revealed the presence of a PED in 16 eyes (84%) at baseline and in 14 eyes (73%) at the last follow-up, with no statistical difference between the two time points (*p* = 0.811) (Table 2). At the last follow-up visit, we found a resolution or decrease of all types of fluid in the majority of eyes. In particular, IRF completely resolved in nine (75%) and reduced in three (25%) of the 12 eyes; SRF completely resolved in 14 (82%) and reduced in three (18%) of the 17 eyes (Figures 1, 2); PED completely resolved in two (13%) and reduced in five (31%) of the 16 eyes, while remained stable in nine eyes (56%; Table 3). Differences between treatment-naïve and previously treated eyes were not analyzed as the two groups presented a high discrepancy in sample size (four vs 15 eyes).

### Safety

In our sample, we found one ocular adverse event. An 85-year-old female who had previously received 82 injections of aflibercept and 12 injections of ranibizumab was switched to brolucizumab due to refractory SRF. The last ranibizumab injection was delivered 4 weeks prior to the first brolucizumab injection, and she received a total of two injections of brolucizumab. Three days after the second IVT-B, she presented with a mild decrease in visual acuity (from 20/50 right before the IVT-B to 20/63), anterior chamber inflammation and an intermediate uveitis classified as mild vitreitis following the Standardization of Uveitis Nomenclature (SUN) Working Group criteria (vitreous haze on dilated fundus microscopy with the details of the posterior pole slightly hazy).(Nussenblatt et al., 1985; Jabs et al., 2005). FA and ICGA were performed excluding retinal vasculitis with or without retinal vascular occlusions and a diagnosis of intraocular inflammation was made. Treatment with prednisolone 1% eyedrops six times daily and 50 mg of oral prednisolone daily was started. After 1 week, the vision remained stable at 20/50 and the symptomatology gradually improved. Steroid treatment was then tapered over a period of 30 days, the inflammatory signs completely resolved without any residual vision loss and the patient was switched back to ranibizumab injections with no further adverse events. An individual case safety report (ICSR) was submitted to the Swiss regulatory authority (SwissMedic). We did not find any other previously described systemic or ocular adverse event.

## Discussion

In the present study, we reported our short-term real-life clinical experience with using intravitreal brolucizumab for the treatment of nAMD patients. At last follow-up, all eyes in this cohort showed a decrease of the CMT with mostly a complete resolution of IRF and SRF and a stabilization of the BCVA. In the registration trials HAWK and HARRIER, brolucizumab showed a greater fluid resolution and a non-inferior visual function in comparison to aflibercept, which were maintained until week 96 (Dugel et al., 2020; Dugel et al., 2021). The results of the present study confirm the high efficacy of brolucizumab in achieving improved anatomical outcomes in nAMD patients, especially in reducing the fluids in the different retinal compartments (SRF, IRF, PED), and consequently, macular thickness. This is remarkable if we consider that the majority (79%) of our patients presented nAMD refractory to other anti-VEGF agents (Figure 2). In particular, fluid reduction was particularly remarkable in the SRF compartment, which reached a statistical significance (*p* = 0.011) at the last visit compared to baseline. The reduction magnitude of IRF and PED size was overall positive even though it did not reach statistical significance (*p* = 0.065 and *p* = 0.811, respectively). None of the eyes showed a deterioration of their anatomical condition at baseline in terms of any of the study parameters.

Visual acuity remained stable despite the anatomical improvement, and the change in BCVA pre-IVT-B and at the last follow-up was not statistically significant (*p* = 0.778).This may be attributed to the fact that most of the eyes in our cohort presented a longstanding history of nAMD with chronic intra- and sub-retinal fluid accumulation that might limit the potential for visual recovery. Moreover, the mean number of previous anti-VEGF injections in the majority of the enrolled eyes was 32, with only four treatment-naïve eyes, while the HAWK and HARRIER trials included selected treatment-naïve eyes only, with a relatively good visual acuity (mean baseline BCVA of Snellen 20/63) (Dugel et al., 2021). Eyes with a baseline BCVA lower than Snellen 20/400, or with fibrosis and/or GA present affecting the central subfield were also excluded from these trials (Dugel et al., 2020). In addition, the follow-up period of our study was too short to reveal an effective improvement in the visual function of our patients and also the post-hoc power analysis (7.2%) revealed a scarce sample size. Nevertheless, our results are comparable to those of the BREW study (Sharma et al., 2021), which was also conducted over a short follow-up period, and included a previously treated patient sample. Also in this study, change in visual acuity following brolucizumab treatment, even if it showed some improvement, did not reach statistical significance (Sharma et al., 2021).

The presence of fluid is a hallmark of disease activity and therefore retinal fluid control is the aim of nAMD management. So far, brolucizumab has shown encouraging results in reducing the presence or amount of fluids in the different retinal compartments (Enríquez et al., 2021). In this context, early real-life experiences with brolucizumab are in accordance with our results showing improvement of anatomical characteristics visible on OCT. In particular, Bulirsch et al. (2021) and Avaylon et al. (2020) showed beneficial OCT outcomes at the one-month visit after the first brolucizumab injection in a cohort of 63 eyes and six patients respectively. Recently, Haensli et al. (2021) reported functional and anatomical improvement at 6-month follow up in seven eyes insufficiently responding to previous anti-VEGF agents. However, it is reasonable to question the impact of retinal fluids on the visual acuity of the patients. This aspect still presents controversy. In fact, according to the current paradigm, the goal of anti-VEGF treatments is to keep the retina as dry as possible, with a zero-tolerance approach to retina fluids (Schmidt-Erfurth et al., 2014), and retreatment strategies such as the treat and extend approach aim to prevent recurrences (Rufai et al., 2017; Silva et al., 2018). Some recent findings, however, paradoxically correlated the presence of SRF with better visual acuity as compared to a dry macula, hypothesizing a protective effect of SRF to vision-threatening macular atrophy (Guymer et al., 2019; Jaffe et al., 2019; Siedlecki et al., 2020). Moreover, the complete resolution of retinal fluids could also increase the long-term incidence of geographic atrophy (Grunwald et al., 2014; Grunwald et al., 2017; Sadda et al., 2018). On the other hand, the results of the HAWK and HARRIER trials highlighted also the importance of fluctuations in the amount of fluids (Sharma et al., 2020a). In these trials, higher central subfield thickness variability, as an indicator of higher fluid fluctuation, was associated with lower BCVA gains up until 96 weeks, while a more stable CMT was associated with both better visual outcomes and a fluid-free retina (Dugel et al., 2021). A recent post-hoc analysis confirmed the importance of fluid control by reducing retinal thickness fluctuations. In particular, eyes treated in the CATT and IVAN studies that presented greater fluctuation in retinal thickness were associated with lower BCVA and a higher risk of developing GA and fibrosis compared to eyes that had less fluctuation (Evans et al., 2020). In this respect, brolucizumab seems promising, but further long-term studies are needed to completely assess its impact on visual function.

In our cohort, one patient developed vitritis 3 days after the second IVT-B. The IOI was diagnosed promptly and managed using high-dose topical and oral corticosteroid therapy with complete resolution without vision loss. Overall, the safety profile of IVT-B in our cohort is in accordance with the post-hoc analysis of the study HAWK and HARRIER, where in eyes treated with 6 mg brolucizumab, the incidence of IOI reported by the investigators was at least 4.6%, with the 48% of the IOI occurring within the first 3 months (Monés et al., 2020). Several case reports described intraocular inflammation (IOI), retinal vasculitis and/or retinal artery occlusion associate with severe visual acuity loss within the first 3 months after IVT-B (Baumal et al., 2020; Kondapalli et al., 2020; Sharma et al., 2020b; Maruko et al., 2021). Therefore, the ophthalmic community is aware of these potential severe adverse events and a post-marketing reporting system actively collects the AEs and will help to determine the figures of this concern in real-life clinical practice (www.brolucizumab.info). Although the pathogenesis of these AEs has not been established with sureness and is currently under investigation (Sharma et al., 2020c; Kondapalli et al., 2020), current recommendations include active surveillance of brolucizumab patients, prompt investigation of every patient experiencing floaters or ocular discomfort persisting for more than 2 days after IVT-B and also of patients presenting with vision loss or light sensitivity at any time following brolucizumab injection (Baumal et al., 2020a). Routine monitoring should be more comprehensive than in standard clinical practice, with special attention to detecting the clinical findings of IOI, retinal arteritis and retinal occlusive events. Wide-field imaging of the retinal periphery is advised to detect any form of inflammation, leakage and/or ischemia (Baumal et al., 2020a).

Our study presents limitations and strengths. First, the small sample size, the short follow-up period, and its retrospective design limit the outcomes of our study. The aim or our investigation was to report the early outcomes of the clinical use of the new drug brolucizumab, hence it was conducted over a relatively small cohort of patient with a short follow-up period, limiting the power and quality of our outcomes. The differences between treatment-naïve and previously treated eyes were not analyzed as presented a high discrepancy in sample size. The eyes included in this study represented the first patients treated at our center with this new molecule starting on March 2020. Therefore in the switch group, we recruited mostly eyes refractory to other anti-VEGF treatments with persistence of retinal fluid despite the regular and 4–6 weeks interval intravitreal treatments, as shown by the high number of previous injections. Moreover, 9 out of 19 included eyes (47%) presented baseline low vision and in 5 (26%) of them the presence of atrophic and/or fibrotic changes or amblyopia limited the potential for visual recovery. However, even in these eyes with chronic lesions, refractory to other anti-VEGF drugs, the good anatomical response to IV-B with complete resolution of fluid resulted in a stabilization of visual acuity and a subjective improvement.

On the other side, we believe that these results represent indeed a strength of this study. In fact, the heterogeneous population enrolled, characterized by a wide spectrum of baseline clinical features and duration of the disease, represents a real-life situation that differs from the selected patients in clinical trials and therefore, it reflects the treatment outcomes of a real clinical practice. To our knowledge, this is one of only a few reports so far on real-life outcomes of intravitreal brolucizumab for the treatment of nAMD. For this reason, we believe that it is of interest to the ophthalmic community and the pharmacovigilance system. Of course, reports on larger cohorts and longer follow up are needed to support the preliminary real-life experiences of this treatment.

In conclusion, in our early real-life experience, brolucizumab resulted highly effective in restoring the anatomy of eyes affected by nAMD and in stabilizing the visual acuity of both treatment-naïve eyes and of eyes refractory to other anti-VEGF intravitreal agents. Nevertheless, the safety profile of this new anti-VEGF treatment should be evaluated carefully and needs further investigations.

## Data Availability

The raw data supporting the conclusion of this article will be made available by the authors, without undue reservation.
